# Short‐Term Urinary Incontinence After Radical Prostatectomy Is Still Based on Patients' Age, Nerve‐Sparing Approach, and Surgical‐Experience, Despite the Higher‐Use of Robotic Surgery in 2022 Compared to 2016 Real‐World Results of a Large Rehabilitation Center in Germany

**DOI:** 10.1002/cnr2.70092

**Published:** 2024-12-28

**Authors:** Lukas Püllen, Max Naumann, Ulrich Krafft, Felix Püllen, Osama Mahmoud, Mulham Al‐Nader, Christopher Darr, Hendrik Borgmann, Christoph Briel, Boris Hadaschik, Johannes Salem, Timur Kuru

**Affiliations:** ^1^ Department of Urology University Hospital Essen Essen Germany; ^2^ Department of Anesthesiology St. Augustinus Hospital Düren Germany; ^3^ Department of Urology, Qena Faculty of Medicine South Valley University Qena Egypt; ^4^ Department of Urology, Faculty of Health Sciences Brandenburg Brandenburg Medical School Theodor Fontane Brandenburg an der Havel Germany; ^5^ Department of Urology Klinik am Kurpark Bad Wildungen Germany; ^6^ CUROS Urologisches Zentrum Klinik Links vom Rhein Cologne Germany

**Keywords:** early results, radical prostatectomy, rehabilitation, urinary incontinence

## Abstract

**Background:**

Despite constant improvements, incontinence is one of the most relevant and quality‐of‐life‐reducing side effects of radical prostatectomy (RP) and, in addition to patient‐specific factors such as age, the experience of the surgeon/center and the surgical technique used play an important role.

**Aims:**

To present current real‐world data on short‐term incontinence after RP from one of the largest German rehabilitation centers in 2022 and to compare it to the results from the same institution in 2016.

**Methods and Results:**

Retrospective, unicentric, univariate analysis of data from 1394 men after RP in 2022 on admission and discharge from the rehabilitation clinic. Incontinence defined as ≥ 1 pad/day was evaluated by quantitative measuring all day incontinence under a defined graduation and compared to the results of 2016. Totally, 1393 men were available for analysis in 2022 compared to 1390 in 2016. Median age for both cohorts was 66 years with minor differences in preoperative PSA levels. Despite different surgical approaches, no significant change in short‐term incontinence rates in 2016 and 2022 were noted at discharge (76.9% vs. 77.9%, *p* = 0.56). A notable increase in patients with ISUP grade Group 2 and a shift towards robotic surgery were observed in 2022 (45.5%–71%). While nerve sparing led to a significant improvement in continence (*p* < 0.01), lymphadenectomy and T‐stage were not related to any significant increase in short‐term incontinence rates. Comparing age groups within the cohort, patients > 69 years exhibited the highest risk of short‐term incontinence and least likelihood of regaining continence during rehabilitation (*p* < 0.01). Men treated at a certified prostate cancer center had significantly (*p* < 0.01) lower short‐term incontinence rates.

**Conclusion:**

Our study shows little improvement in short‐term postoperative incontinence rates after RP in Germany in the last 6 years and known risk factors for postoperative incontinence like age, nerve‐sparing surgery, and level of experience were reproduced in our analyses. We conclude not only to carefully select but also to counsel patients before being treated for prostate cancer and to strongly advice treatment at certified centers.

## 1 Introduction

Incontinence is a common side effect after radical prostatectomy (RP) and has a major impact on quality of life and healthcare costs [1]. One year after surgery, incontinence rates range from 20% (one pad change in 24 h) to 57% (not pad and not leakage free) [2] and on the other hand, “no pad continence” is only present in around one in 10 men [3]. In addition to patient‐specific factors such as age or body mass index (BMI), the surgeon's experience and the technique used play a significant role, with the robot‐assisted method tending to show better results [3, 4, 5]. In this context, nerve‐sparing technique can lead to better continence rates [6], whereby the technical aspect and less the preservation of the neurovascular bundle seems to have the decisive influence here [7]. The increasing use of robotic surgery has also raised expectations with regard to (functional) outcomes, which must be taken into account during the pre‐therapeutic consultation [8], especially when it comes to sexual function [9]. In Germany, inpatient rehabilitation is an essential part of perioperative care. Real‐world data from one of the largest German urological rehabilitation clinics from 2009 and 2016 have already been presented in the past showing short‐term continence of 33.9% (2009) and 23% (2016), respectively [10, 11]. The aim of our work was to show novel data from 2022 and to compare it with 2016 in order to map the reality of care for incontinence as one of the most relevant side effects of RP.

## 2 Materials and Methods

In this retrospective and monocentric study data of patients who received inpatient follow‐up treatment after RP for prostate cancer at the Klinik am Kurpark in Bad Wildungen‐Reinhardshausen in 2022 were evaluated for short‐term incontinence status recorded at the time of admission to the rehabilitation clinic, during treatment and at the time of discharge. These data were compared with the already evaluated and published [10] results of the year 2016 from the same institution. Therefore, we chose the same approach when evaluating the actual data. The study was conducted in accordance with the ethical standards of the Declaration of Helsinki and approved by the local ethics committee (24‐12 262‐BO).

The status of urinary incontinence was determined by the continuous 24‐h pad test according to a standardized classification (Wildunger Stadieneinteilung). The extent of urine loss was divided into three grades as in the publication from 2022 and presented in an incontinence formula (Appendix A) [10]. Incontinence was defined as ≥ 1 pad/day as this is a widely used definition in other studies.

Since men over 70 years of age are more likely to develop incontinence after RP [12], the age groups were defined as follows: < 60, 60–69 and > 70 years.

The statistical calculations were performed using the statistical software R (open source, founder Ross Ihaka and Robert Gentleman 1992 University of Auckland, New Zealand), an open‐source software with the use of descriptive statistics, the *Χ* ^2^ test, Student's *t*‐test and Fisher exact test when calculating odds ratios (OR). In line with the preliminary work by Briel et al. on the 2016 data [10], our analysis was also univariate. Confounding variables were not addressed in our analysis.

Patients were only excluded from the analysis when the surgical technique was not recorded.

## 3 Results

Among all patients that received treatment at Klinik am Kurpark in 2022, 1393 underwent RP and were thus assessable for analysis. This data was compared to the results from the analysis of 1390 patients treated in 2016 at the same institution. Clinical characteristics of patients undergoing urological rehabilitation post RP in 2016 and 2022 are presented in Table 1. Median age for both cohorts was 66 years with minor differences in preoperative PSA levels. However, a notable increase in patients with ISUP 2 (42% vs. 50.2%), and a shift toward robotic‐assisted RP (RARP) were observed in 2022 (45.5% vs. 71%).

**TABLE 1:** Comparison of the patient cohorts from 2016 and 2022 with regard to age, preoperative PSA values, postoperative tumor stages and Gleason score, as well as the surgical procedures performed.

	2016 (*n* = 1390)	2022 (*n* = 1393)	*p*
	Median (IQR)		
Age (years)	66 (61–71)	66 (61–71)	0.86
Preoperative PSA (ng/mL)	8 (5.6–12.4)	7.56 (5.3–11.6)	0.03
Postoperative T‐stage (*n*)			
pT0‐pT2c	855 (61.4%)	910 (65.4%)	< 0.01
pT3a‐pT4	507 (36.6%)	450 (32.3%)	
pTx	28 (2.0%)	33 (2.3%)	
ISUP grade groups (*n*)			
ISUP 1	128 (9.5%)	97 (7%)	< 0.01
ISUP 2	581 (42%)	698 (50.2%)	
ISUP 3	329 (24%)	334 (23.9%)	
ISUP 4	146 (11%)	92 (6.6%)	
ISUP 5	181 (13%)	138 (9.9%)	
NA	25 (0.5%)	34 (2.4%)	
Surgical procedures (*n*)			
EERPE	51 (3.7%)	30 (2.2%)	< 0.01
RARP	633 (45.5%)	989 (71%)	
RPE	687 (49.5%)	363 (26%)	
RPP	19 (1.3%)	11 (0.8)	

Abbreviations: EERPE: endoscopic extraperitoneal radical prostatectomy; RARP: robotic‐assisted radical prostatectomy; RPP: radical perineal prostatectomy; RRP: (open) radical retropubic prostatectomy.

**FIGURE 1:**
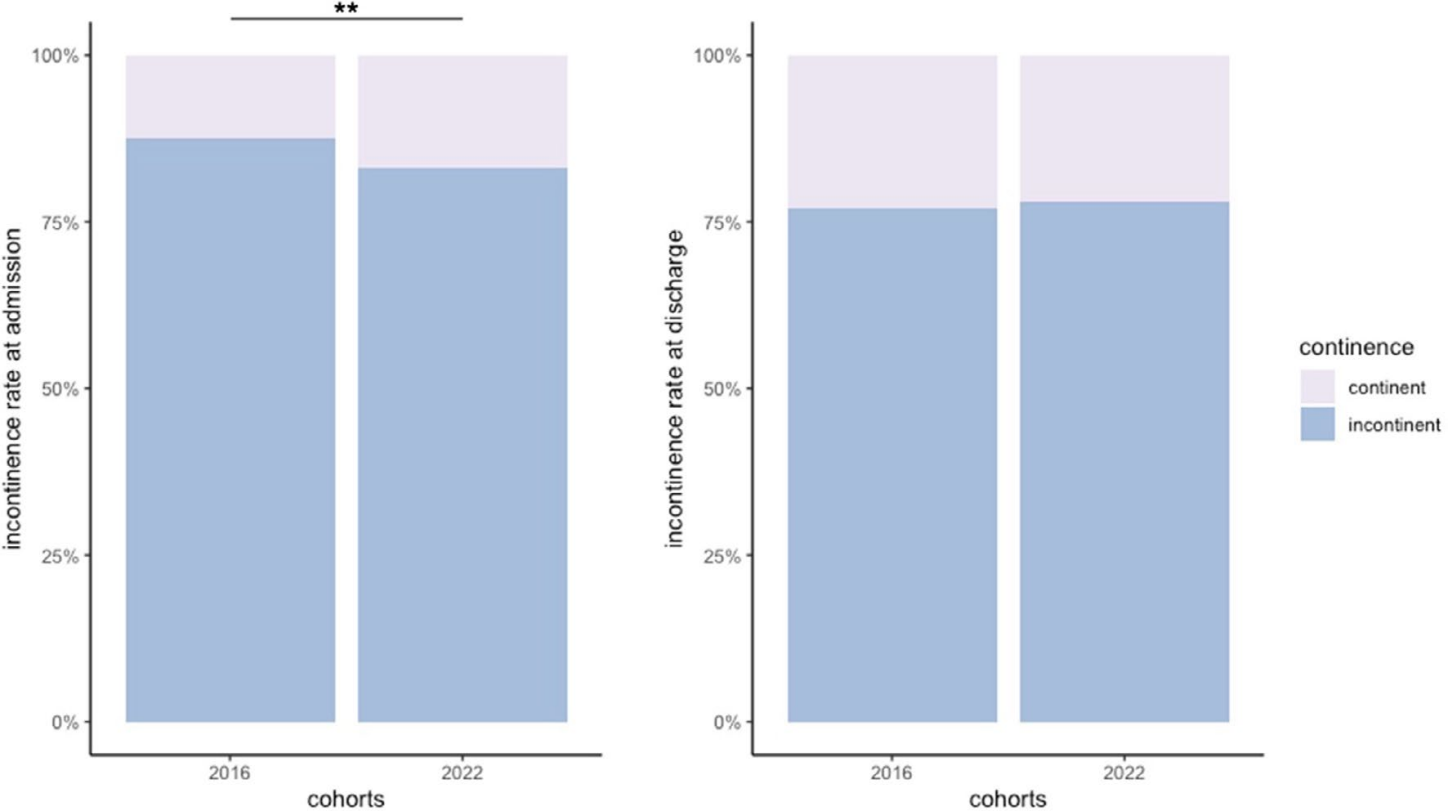
Comparison of incontinence on admission and discharge in 2016 and 2022. **p < 0.05.

**FIGURE 2:**
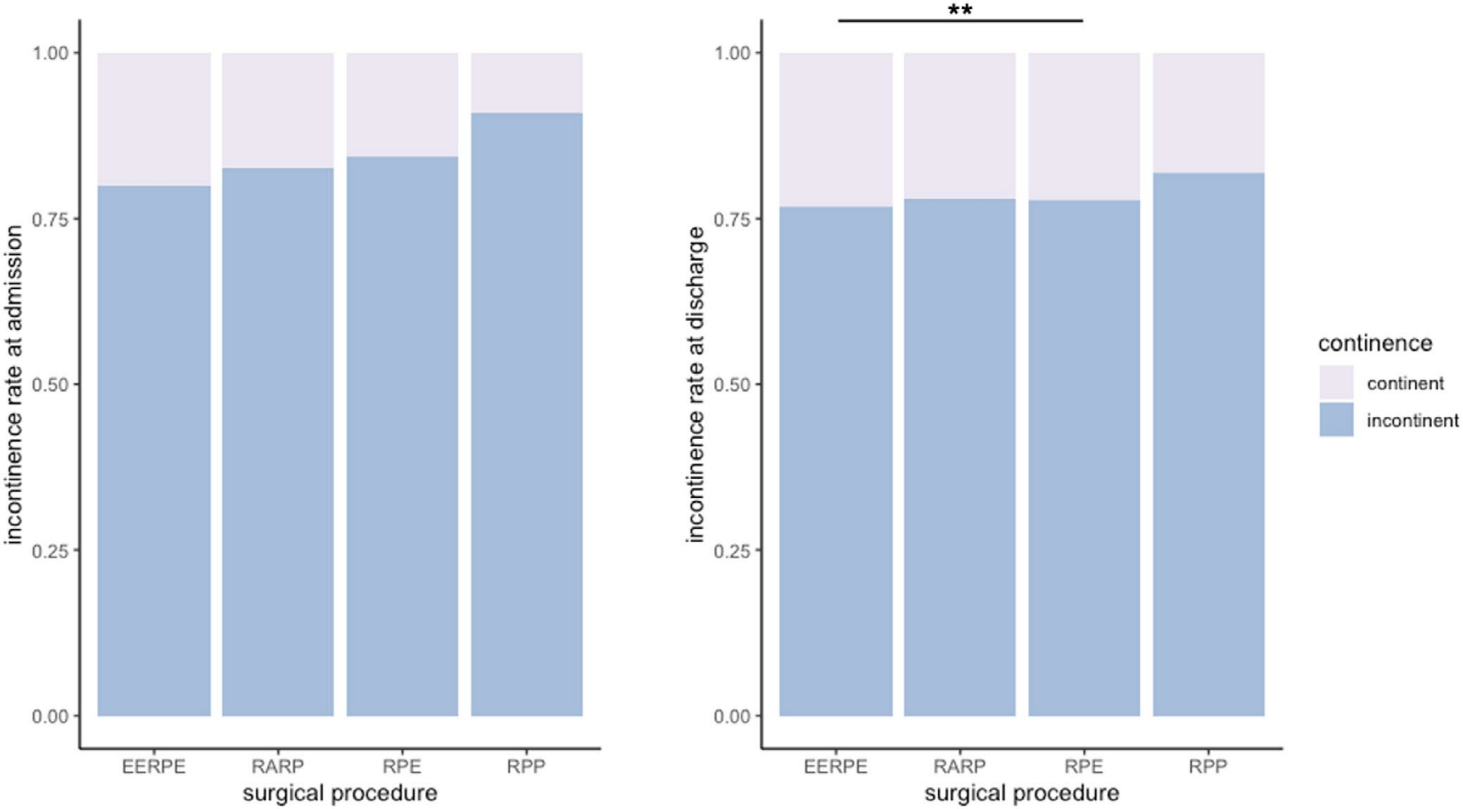
Incontinence depending on the surgical technique on admission and discharge in 2022. EERPE: Endoscopic extraperitoneal radical prostatectomy; RARP: Robotic‐assisted radical prostatectomy; RPP: Radical perineal prostatectomy; RRP: Radical retropubic prostatectomy. **p < 0.05.

Despite different surgical approaches, no significant change in short‐term incontinence rates in 2016 and 2022 were noted at discharge from urological rehabilitation (76.9% and 77.9%, *p* = 0.56). However, a slight improvement in early continence rates in 2022 was observed upon admission (12.6% vs. 16.9%, *p* < 0.01) (Figure 1). Analysis of subgroups in 2022 revealed open retropubic prostatectomy had a significant benefit over a laparoscopic approach at discharge (*p* < 0.05), albeit with a modest effect and low laparoscopic cases. Other surgical methods showed no significant differences in incontinence rates (Figure 2).

**FIGURE 3:**
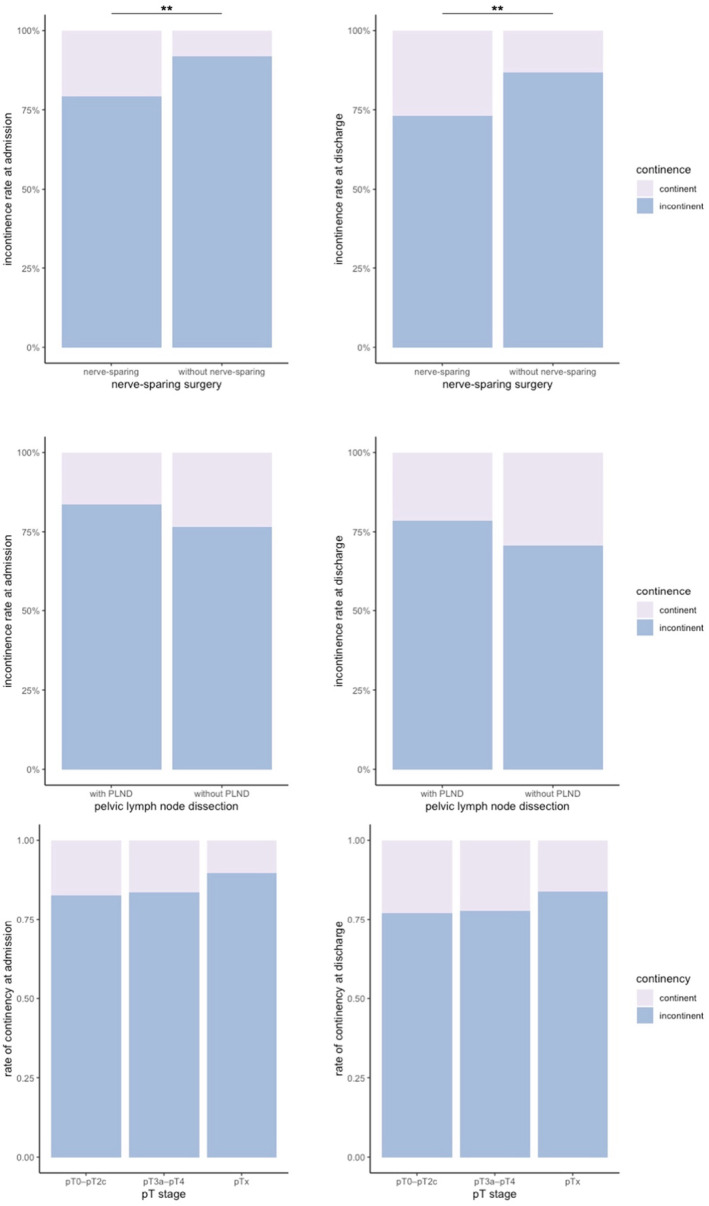
Comparison of incontinence on admission and discharge in 2022 depending on nerve‐sparing, pelvic lymph node dissection and T‐stage. **p < 0.05.

Nerve‐sparing surgery demonstrated significantly lower short‐term incontinence rates compared to non‐nerve‐sparing surgery (*p* < 0.01; OR 0.42 CI 0.28–0.62). Conversely, the presence of lymphadenectomy did not significantly impact short‐term incontinence rates. Tumor T‐stage showed a slight and statistically significant shift toward localized prostate cancer (Table 1) but did not significantly affect short‐term incontinence (*p* = 0.57) (Figure 3).

Of the patients who received nerve sparing, the majority (74.6%, *p* < 0.01) were in lower tumor stages (pT0‐pT2c).

**FIGURE 4:**
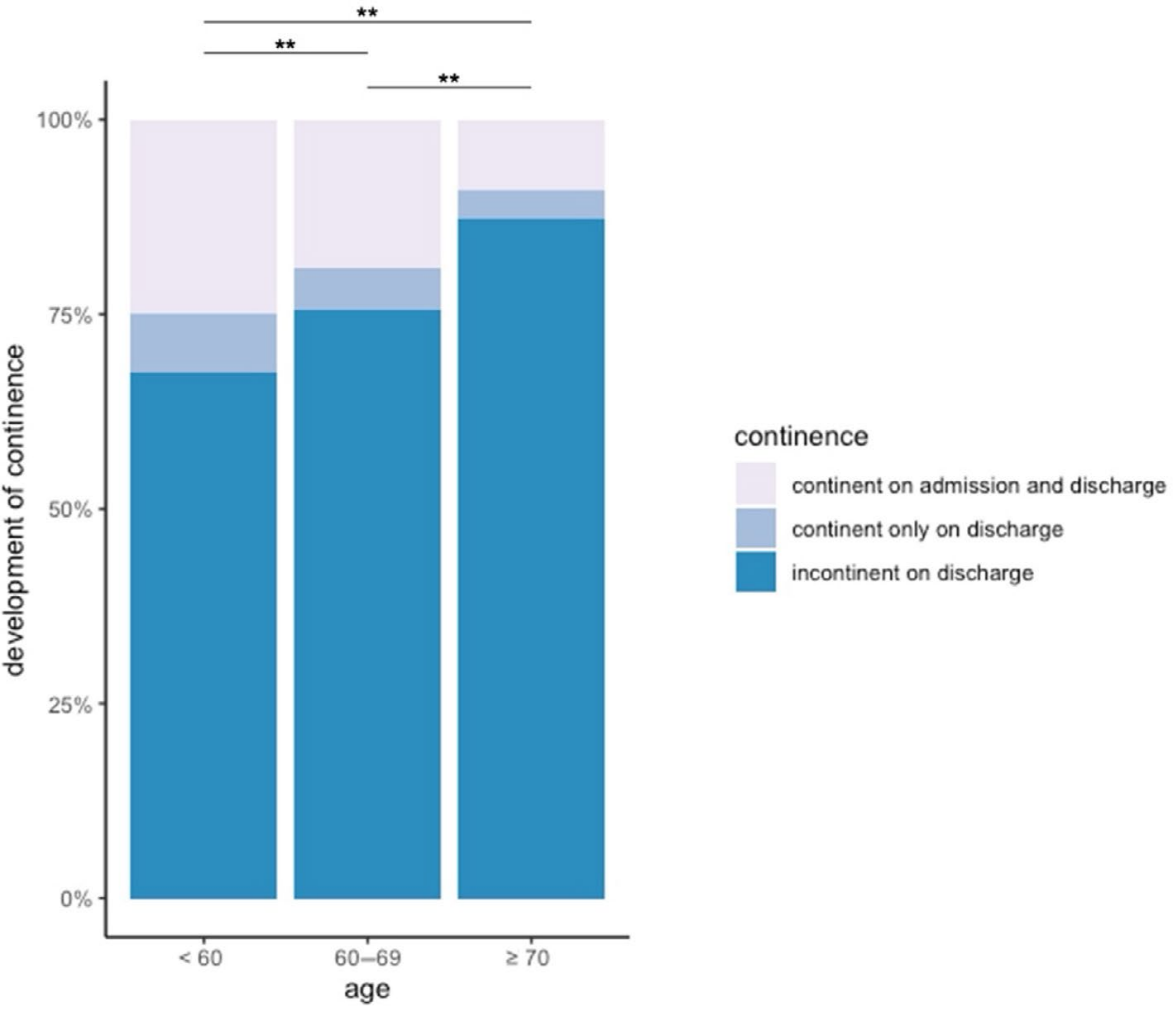
Comparison of incontinence on admission and discharge between age groups in 2022. **p < 0.05.

Comparing age groups within the cohort, patients over 69 years exhibited the highest risk of short‐term incontinence and least likelihood of regaining continence during rehabilitation (*p* < 0.01). Age significantly influenced continence rates at admission and discharge (*p* < 0.01) (Figure 4).

**FIGURE 5:**
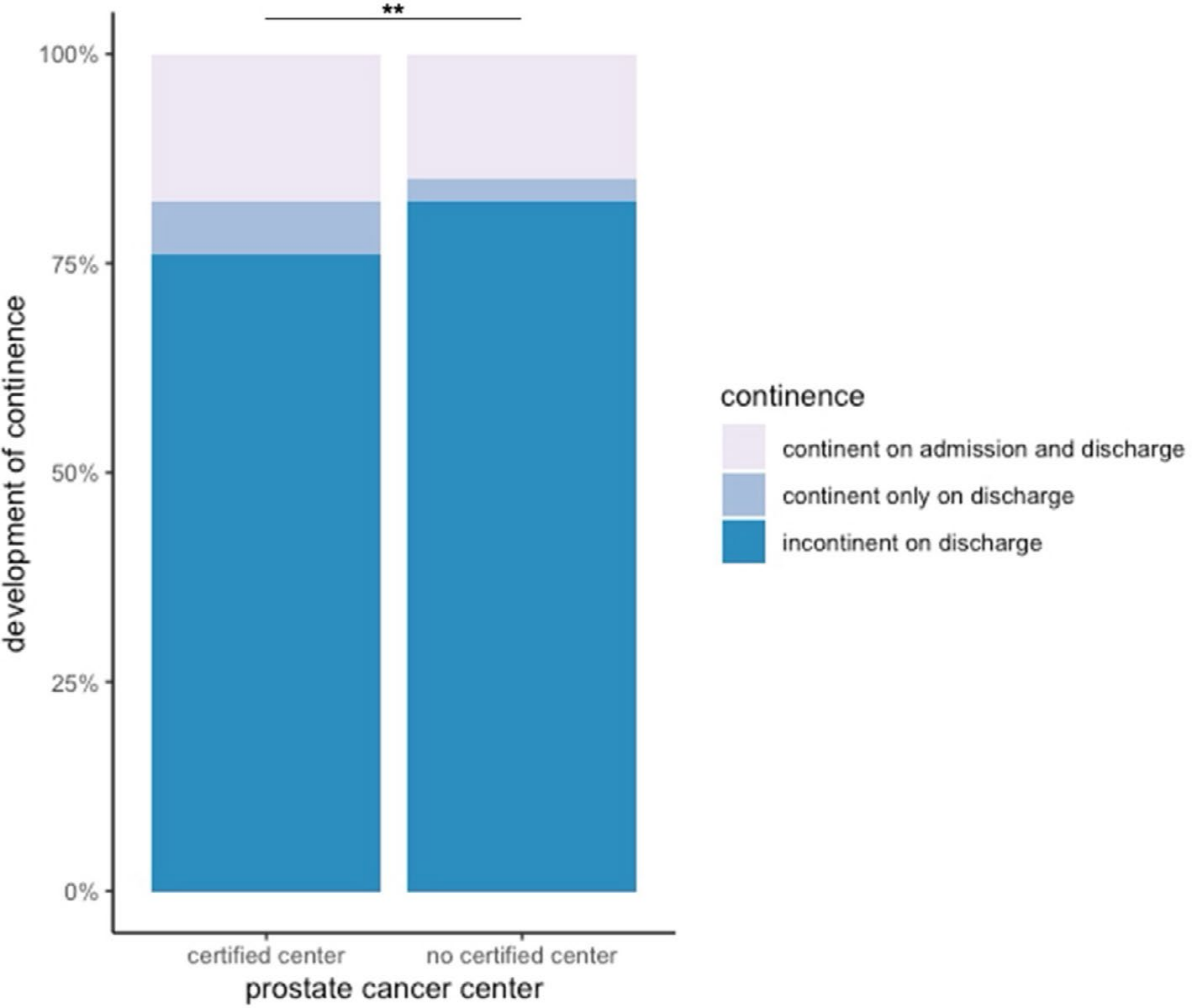
Comparison of incontinence on admission and discharge between certified and non‐certified centers in 2022. **p < 0.05.

Out of all patients, 893 were treated at a certified prostate cancer center (DKG, German Cancer Society) before urological rehabilitation admission. In this context, certified means that the clinics must meet certain quality indicators, a certain number of cases and demonstrate compliance with them in annual inspections. Significant differences in short‐term incontinence rates were observed at admission and discharge (*p* < 0.01, OR 0.69 CI 0.50–0.93) leading to rates 6% lower for patients that received surgical treatment at a certified prostate cancer center (Figure 5).

The exact time interval between surgery, admission and discharge at the rehabilitation clinic was not recorded. In Germany, patients usually start their rehabilitation 2 weeks after discharge following surgery, which usually lasts 3 weeks.

## 4 Discussion

The aim of this study was to show the treatment reality for short‐term incontinence after RP over the past years in Germany with our data showing that despite constant improvements in rehabilitation as well as in surgical methods, a relevant number of men treated in the initial period continue to suffer from incontinence. Age is a significant risk factor for incontinence and frequency at certified centers improves continence [13, 14]. In future, we should therefore focus not only on how to improve continence after RP but also on how to prepare our patients for possible incontinence and how to assess even more rigorously which patients should be treated and how. Against the background of a decision regret in almost 20% of men undergoing local therapy for organ‐confined tumors [15], these points are increasingly coming into focus, especially if shared decision‐making can reduce the odds by up to 40% [16] with urinary continence being one aspect lowering decision regret in the HAROW‐trial [17]. The surgical approach was not associated with (intermediate term) decision regret, but patients treated with RARP reported more active participation in decision‐making [18]. The use of predictive nomograms could help both the physician and the patient to make a better and more individualized decision [19, 20, 21] and with an ever‐increasing acceptance of digital decision aids and the demand for a transparent information process, this could enable a less emotional and eminence‐based discussion. At a time when shared decision‐making is increasingly demanded, this approach could also support physicians in taking a critical stance (e.g., against surgery in the case of severe obesity despite the patient's personal preference) and increase both the acceptance of such advice and the consequences of therapy in general.

Although this paper deals with the effects of RP, it should not go unmentioned in this context that forms of radiotherapy can also lead to incontinence, albeit less frequently and to a lesser extent than with R [22, 23, 24]. Ultimately, no statistics can replace a critical and honest dialog with our patients, which in the case of prostate cancer treatment must also include the possibility of permanent incontinence, but also its available and effective treatment options (e.g., sub‐urethral slings).

We would therefore like to emphasize that the incontinence rates shown only reflect the short‐term period and that the early evaluation had an influence on this, which is supported by the results of other studies: 69.6%–87.7% after 1 month [25, 26, 27].

As reported before by Coughlin et al. [5] the surgical approach (open vs. robotic) does not transfer into improved continence between the several techniques. The study by Coughlin et al. analyzed data between 2010 and 2014, while our study includes data from 2016 to 2022. Despite the steady increase in experience and further development of RARP, this also shows the limitations. Higher technical resolution, number of robotic arms, freedom of movement and improved visualization seems not to result in better continence rates. This fact should therefore be discussed with patients as part of a realistic patient education process to avoid generating excessive expectations through the sole use of the robot.

Nerve sparing was a significant predictor of short‐term incontinence compared to non‐nerve sparing. These findings are consistent with current studies that show positive effects on the development of postoperative continence, particularly in the case of bilateral nerve sparing [6, 28, 29]. However, it should be borne in mind that these effects are more likely to apply in the case of good preoperative sexual function, which was not recorded in our analysis [6].

Although the T‐stage was not significantly associated with short‐term continence in our analysis, an influence can at least be indirectly inferred, as a lower tumor stage was significantly more frequently associated with nerve sparing. One reason for this could be the steadily expanding role of multiparametric MRI, which enables a better assessment of the local, clinical T‐stage [30] and thus the planning of a nerve‐sparing procedure. This statement is subject to the proviso that we have no information on the presence of an MRI in the patients analyzed.

Our study shows that continence rates get significantly worse with increasing age. Older (65–75 years) or elderly (> 75 years) men feel significantly more burdened by their postoperative continence situation and, in contrast to younger men (96%–98%), are significantly less likely to reach the stage of social continence (85%–92%), defined as max. 1 pad/day [14]. Therefore, geriatric assessment prior treatment decision has a high impact on life quality as frail and old patients should be avoided for radical surgery. Current data from 125 certified centers collected as part of the prostate cancer outcome (PCO) study (between 2016 and 2022) also show that around 43% of patients require at least 1 pad/day 1 year after surgery. Using the domain score for incontinence from the EPIC‐26 (expanded prostate cancer index composite), the self‐reported data of 17 149 men were examined and 97% of these men reported no pad use before surgery. The age groups 70–79 and over 79 years did report higher rates of pad use after surgery (52.3% and 68%, respectively). These results are in line with our findings showing significantly higher incontinence rates for patients older than 70 years and lower improvement of continence between admission and discharge.

Our data show a significantly lower short‐term incontinence rate of 6% in patients who underwent surgery at a certified center. An evaluation of more than 22 000 German patients from 2008 to 2017 also showed improved incontinence rates for the certified centers [13]. Another German study group was able to show this effect at least for robotically assisted prostatectomy, which is offered more frequently at certified centers [31]. Overall, however, these results should be evaluated carefully, as certifications primarily address oncosurgical aspects and surgeon experience [32].

In our study, preoperative pelvic floor training was not recorded, which must generally be seen as a limitation. In contrast, however, it can be stated that although the effect of pelvic floor training is significant 3 months postoperatively, in the short (1 month) and in longer term (6 months) effects were not relevant [33].

No distinction was made between unilateral and bilateral nerve sparing, which must also be considered a limitation, as there are significant differences in terms of postoperative continence [28, 29]. In our study, only a short follow‐up period was presented, which does not allow any conclusions to be drawn about long‐term quality of life and functionality. On the other hand, as already mentioned, our data show that patients must be encouraged to continue working and remain motivated. The additional use of medical applications (apps) may have a significant effect in longer‐term motivation [34]. In addition to the factors mentioned above, body mass index/obesity was also not recorded and investigated as a possible factor influencing functional outcomes after RP [35, 36], which must also be seen as a weakness. Another important limitation is the retrospective and univariate analysis and the lack of a structured timeline from surgery to admission and discharge at the rehabilitation center. Although the treatment was partially standardized, this is not comparable with a prospective multivariate analysis conducted according to a protocol. Future studies should therefore include patient‐recorded outcomes and quality of life measures (starting before surgery) as well as a standardized timeline including a defined exercise plan in addition to the recording of influencing factors (e.g., preoperative pelvic floor training) and a longer follow‐up period.

## 5 Conclusion

Our study shows little improvement in short‐term incontinence rates after RP in Germany in the last 6 years. Known risk factors for postoperative incontinence like age, nerve‐sparing surgery, and level of experience were reproduced in our analyses. Due to the possible high impact on quality of life by persisting incontinence, we conclude to carefully select and counsel patients before prostate cancer treatment regardless of the modality and surgical technique used and strongly advice treatment at certified centers.

## Author Contributions

**Lukas Püllen:** statistical evaluation, manuscript writing. **Max Naumann:** statistical evaluation, critical reading. **Christoph Briel:** data compilation, critical reading. Johannes Salem: conceptualization. **Timur Kuru:** conceptualization, critical reading. All other authors: critical reading.

## Conflicts of Interest

The authors declare no conflicts of interest.

## Data Availability

The data that support the findings of this study are available on request from the corresponding author. The data are not publicly available due to privacy or ethical restrictions.

## Appendix A

– Continence: I0.
– Incontinence: I1.
– Daytime urine loss (07:00–22:00): D.
– Urine loss at night (22:00–07:00): N.
– Grade 1: loss ≤ 100 mL.
– Grade 2: loss between 100 and 250 mL.
– Grade 3: loss > 250 mL.

Incontinence formula:

$$I0/\text{I1 D1}-3\;\left(\mathrm{mL}\right)\;\text{N1}-3\;\left(\mathrm{mL}\right).$$
